# Supplementary material for: Empirical methods for controlling false positives and estimating confidence in ChIP-Seq peaks
Source: BMC Bioinformatics. 2008 Dec 5;9:523. doi: 10.1186/1471-2105-9-523 (PMC2628906; doi:10.1186/1471-2105-9-523)
Supplement: Additional File 1 — A variety of html documents from the USeq web site detailing the available applications, their best usage, output file type descriptions, command line menus, etc. [file 1471-2105-9-523-S1.zip › USeqUserGuides/JavaDoc.zip!/JavaDoc/bioroot/TableHeading.html]

TableHeading


|  |  |  |
| --- | --- | --- |
| **Overview** \| **Package** \| **Class** \| **Use** \| **Tree** \| **Deprecated** \| **Index** \| **Help** | |  |
| **PREV CLASS**   **NEXT CLASS** | **FRAMES**    **NO FRAMES**     **All Classes** |  |
| SUMMARY: NESTED \| FIELD \| CONSTR \| METHOD | DETAIL: FIELD \| CONSTR \| METHOD |  |


---


## bioroot Class TableHeading

```
java.lang.Object
  bioroot.TableHeading
```

**All Implemented Interfaces:**: java.lang.Comparable

---

``` public class TableHeading extends java.lang.Object implements java.lang.Comparable ```

**Author:**
:   nix

---

**Constructor Summary**

`TableHeading(java.lang.String name,
int originalOrderNumber)`


| **Method Summary** | |
| --- | --- |
| `int` | `compareTo(java.lang.Object obj)`             For sorting |
| `java.lang.String` | `getCheckBoxNumber(java.lang.String groupNameCheckBox)`             Makes a checkbox and textfield where the box is checked if TableHeading is visible and a rounded double orderNumber is put in text field if it's not 1000 |
| `java.lang.String` | `getFullName()` |
| `java.lang.String` | `getName()` |
| `double` | `getOrderNumber()` |
| `double` | `getOriginalOrderNumber()` |
| `boolean` | `isVisible()` |
| `void` | `resetOrderNumber(javax.servlet.http.HttpServletRequest request)`             Sets orderNumber from parameter provided, the Param can be converted to an int that is >0. |
| `void` | `setFullName(java.lang.String string)` |
| `void` | `setName(java.lang.String string)` |
| `void` | `setOrderNumber(double i)` |
| `void` | `setVisible(boolean b)` |
| `java.lang.String` | `toString()` |

**Methods inherited from class java.lang.Object**

`equals, getClass, hashCode, notify, notifyAll, wait, wait, wait`

**Constructor Detail**

### TableHeading

```
public TableHeading(java.lang.String name,
                    int originalOrderNumber)
```


**Method Detail**

### toString

```
public java.lang.String toString()
```

:   **Overrides:**: `toString` in class `java.lang.Object`

---


### compareTo

```
public int compareTo(java.lang.Object obj)
```

:   For sorting

    :   **Specified by:**: `compareTo` in interface `java.lang.Comparable`

---


### getCheckBoxNumber

```
public java.lang.String getCheckBoxNumber(java.lang.String groupNameCheckBox)
```

:   Makes a checkbox and textfield where the box is checked if TableHeading is visible and a rounded double
    orderNumber is put in text field if it's not 1000

---


### resetOrderNumber

```
public void resetOrderNumber(javax.servlet.http.HttpServletRequest request)
```

:   Sets orderNumber from parameter provided, the Param can be converted to an int that is >0.
    Otherwise, no change.

---


### getName

```
public java.lang.String getName()
```

---


### getOrderNumber

```
public double getOrderNumber()
```

---


### isVisible

```
public boolean isVisible()
```

---


### setName

```
public void setName(java.lang.String string)
```

---


### setOrderNumber

```
public void setOrderNumber(double i)
```

---


### setVisible

```
public void setVisible(boolean b)
```

---


### getFullName

```
public java.lang.String getFullName()
```

---


### setFullName

```
public void setFullName(java.lang.String string)
```

---


### getOriginalOrderNumber

```
public double getOriginalOrderNumber()
```


---


|  |  |  |
| --- | --- | --- |
| **Overview** \| **Package** \| **Class** \| **Use** \| **Tree** \| **Deprecated** \| **Index** \| **Help** | |  |
| **PREV CLASS**   **NEXT CLASS** | **FRAMES**    **NO FRAMES**     **All Classes** |  |
| SUMMARY: NESTED \| FIELD \| CONSTR \| METHOD | DETAIL: FIELD \| CONSTR \| METHOD |  |


---
